# Multi-labelled proteins recognition for high-throughput microscopy images using deep convolutional neural networks

**DOI:** 10.1186/s12859-021-04196-3

**Published:** 2021-06-15

**Authors:** Enze Zhang, Boheng Zhang, Shaohan Hu, Fa Zhang, Zhiyong Liu, Xiaohua Wan

**Affiliations:** 1 High Performance Computer Research Center, Institute of Computing Technology, Chinese Academy of Sciences, Beijing, China; 2 University of Chinese Academy of Sciences, Beijing, China; 3 Department of Automation, Tsinghua University, Beijing, China; 4 School of Software, Tsinghua University, Beijing, China

**Keywords:** Protein pattern recognition, DNNs, Multi-class and multi-label, Label imbalance, High-throughput microscopy images

## Abstract

**Background:**

Proteins are of extremely vital importance in the human body, and no movement or activity can be performed without proteins. Currently, microscopy imaging technologies developed rapidly are employed to observe proteins in various cells and tissues. In addition, due to the complex and crowded cellular environments as well as various types and sizes of proteins, a considerable number of protein images are generated every day and cannot be classified manually. Therefore, an automatic and accurate method should be designed to properly solve and analyse protein images with mixed patterns.

**Results:**

In this paper, we first propose a novel customized architecture with adaptive concatenate pooling and “buffering” layers in the classifier part, which could make the networks more adaptive to training and testing datasets, and develop a novel hard sampler at the end of our network to effectively mine the samples from small classes. Furthermore, a new loss is presented to handle the label imbalance based on the effectiveness of samples. In addition, in our method, several novel and effective optimization strategies are adopted to solve the difficult training-time optimization problem and further increase the accuracy by post-processing.

**Conclusion:**

Our methods outperformed the SOTA method of multi-labelled protein classification on the HPA dataset, GapNet-PL, by above 2% in the F1 score. Therefore, experimental results based on the test set split from the Human Protein Atlas dataset show that our methods have good performance in automatically classifying multi-class and multi-labelled high-throughput microscopy protein images.

## Background

**Fig. 1:**
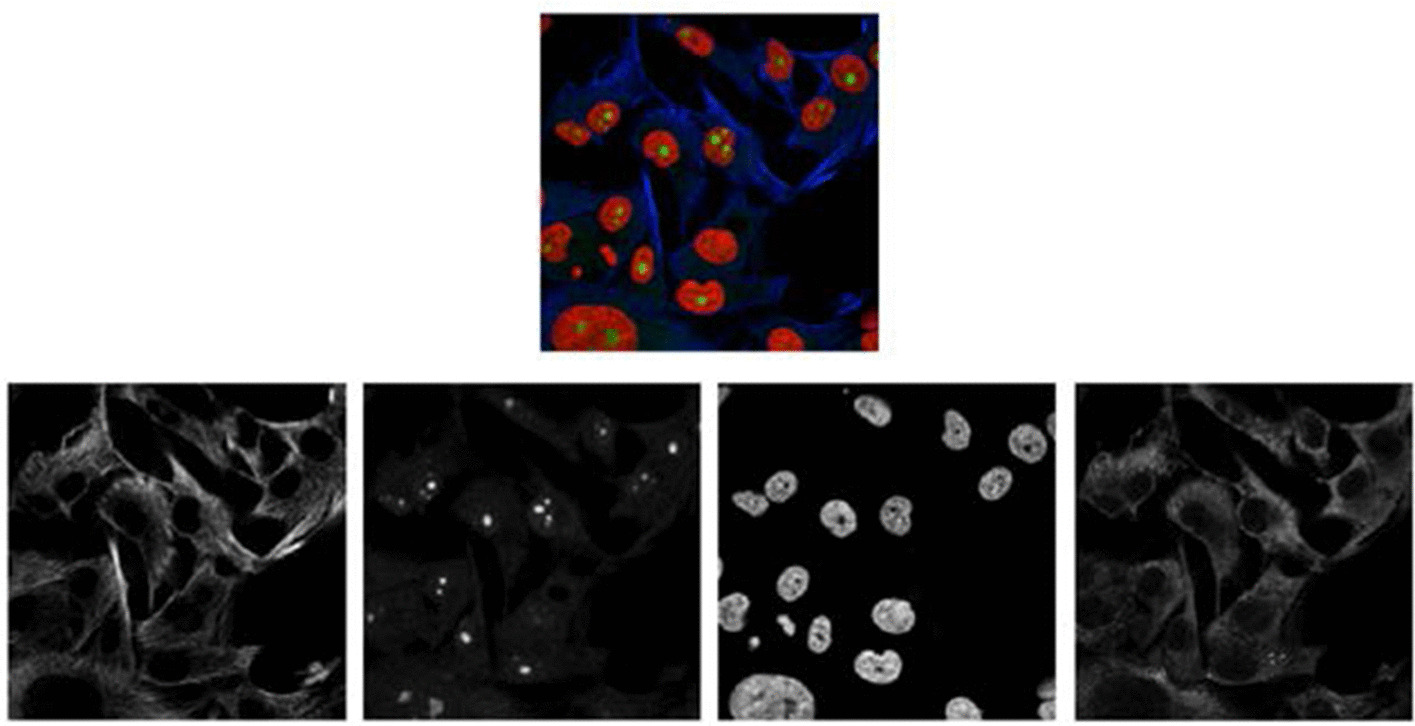
One protein microscopy image from HPA Cell Atlas datasets (above) and the gray images of 4 channels (RGBY) generated from the microscopy image (below)

Proteins execute all kinds of functions in the human body within different types of cells, and proteins in various environments perform differently. Therefore, recognizing and understanding proteins under distinct circumstances are vital to studying the physiological activity of people. Recently, the HPA (Human Protein Atlas) [1] was built as a smart microscopy system to identify and localize proteins through high-throughput images. An algorithm capable of classifying mixed patterns of proteins was needed to make the system “smarter”. Although experts can generally classify protein images manually, the process would be highly time consuming. In addition, there are many similar and confusing patterns and features in these protein images, making this task even more difficult. Therefore, the algorithm or method is expected to accurately and efficiently recognize multi-patterns that are mixed together among various types of cells. As the HPA project has provided adequate protein microscopy images, as shown in Fig. 1, with annotations to feed a large neuron network, we decided to adopt the DNN method, which has good performance in classifying images.

DNNs (deep neuron networks) have become an extremely popular tool in computer vision and image analysis areas. In particular, CNNs (convolutional neuron networks), e.g., InceptionNet [2], ResNet [3], DenseNet [4], have been applied to classification, detection, segmentation, and tracking due to their good feature learning and representation. Recently, CNNs have also been used to analyse microscopy images, such as biological images and medical images [5], such as MIL [6] and M-CNN [7]. However, the effects of these methods are usually unsatisfactory for extremely noisy data. Additionally, the patterns in these data are always complex, and the resolution is high. Therefore, new methods are urgently required to adapt to this kind of dataset.

GapNet-PL [8] is a SOTA architecture designed to tackle the characteristics of high-throughput fluorescence microscopy imaging data. Because the spatial details and context are equally important in biomedical recognition tasks, this architecture extracts and combines some different levels and stages of intermediate features, which perform well on HPA datasets. However, the lightweight backbones of GapNet are too naive to deal with such complex protein datasets well. Meanwhile, the performance will decline in situations of severe label imbalance. We designed a novel data processing method while making training and testing datasets based on the data characteristics.

First, when designing networks, we proposed customized layers such as “buffering” layers and adaptive concatenate pooling (ACP) layers in the base models of the encoder and head parts, which made the proposed networks more adaptive to the task. Additionally, we introduced the hard sampler at the end of the architecture for mining hard examples better. Second, E-loss was proposed based on the effectiveness of samples for each class to solve the label imbalance. Finally, we developed novel optimization strategies in training and testing time. Group learning and cyclic learning can help the models converge better, and the multi-threshold searching process fits the model to the validation and test sets, both improving the accuracy of the proposed methods.

The experimental results demonstrate that our methods make the base models achieve higher F1 scores than other baseline approaches, including the SOTA method. In the following section, we explain our datasets in detail, as well as the methods proposed for improving the model performance.

### Datasets

All experiments were conducted on datasets released for the “Human Protein Atlas Image Classification” challenge by the Human Protein Atlas project [9]. The main dataset contains approximately 30,000 samples for training and 11,500 samples for testing from part of the HPA Cell Atlas led by Dr. Emma Lundberg. There are 28 distinct classes (or types) of proteins in the dataset. The data were collected by the confocal microscopy approach. However, the dataset includes 27 different cell types with highly different morphologies, which could influence the protein patterns of the distinct organelles. Every sample in the dataset is formed from 4 image channels, red, green, blue and yellow. Each channel filter is stored as an individual file. The red channel represents microtubules, green represents proteins, blue represents the nucleus, and yellow represents the reticulum.

**Fig. 2:**
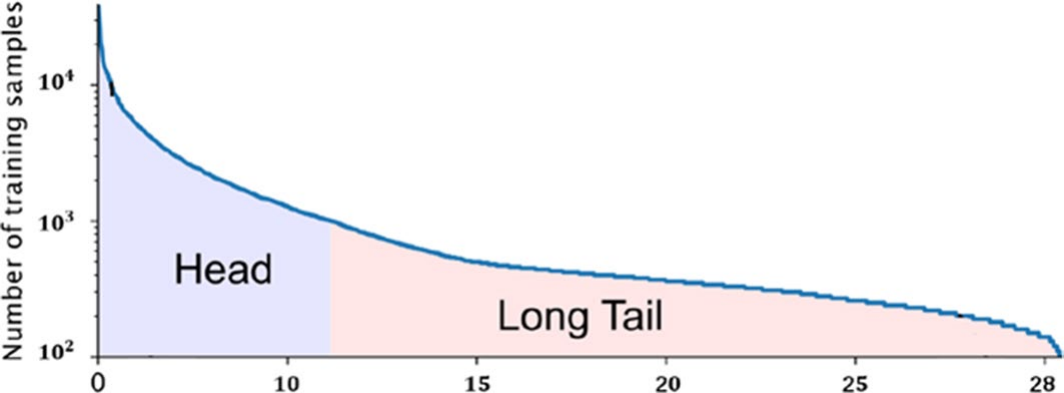
Long tail distribution that all 28 protein classes follow in the whole dataset

Obviously, our classification task is multi-class and multi-label. The number of labels of one sample can range from one to twenty-eight. In addition, as we can see in Fig. 2, there is an extreme label imbalance in the dataset. Some majority types of proteins may take most (several tens of thousands) of the whole dataset, e.g., the nucleoplasm and cytosol. Rare classes, such as rods and rings, can account for only a few hundred, which are difficult to learn but play an important role in the score. Therefore, this imbalance problem urgently needs to be solved. We removed approximately 6000 duplicated samples, which seem extremely similar to image hashing because they are mostly majority classes.

To handle this imbalance problem, we first oversample the minority classes a few times to increase their number. We cannot oversample too much because this can cause overfitting. Then, we adopt multi-label stratification splitting to balance any inconsistent minority distributions that may exist. We split the training/validation data and different training data folds randomly and equally.

**Fig. 3:**
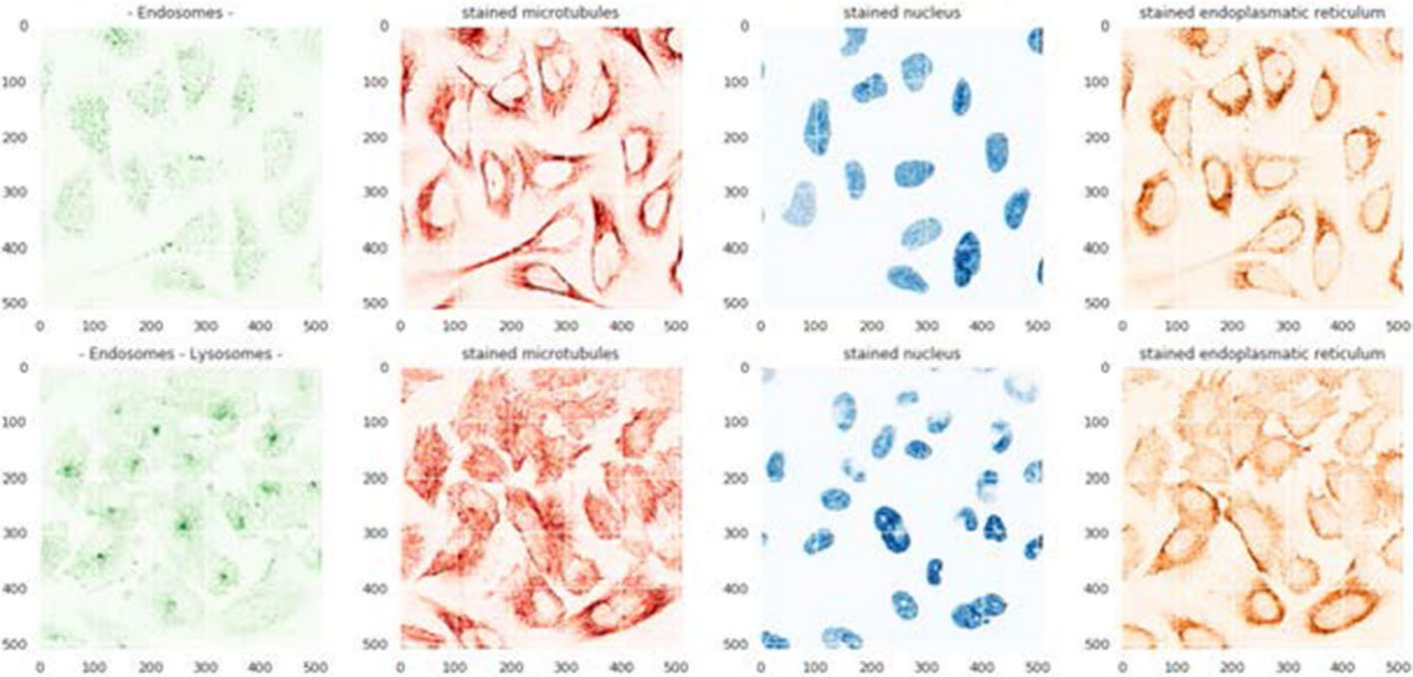
Protein images (green) related to endosomes and three other filter channels. The first row shows a sample that contains only endosomes proteins. The second row displays another sample containing two mixed types of protein including endosomes and lysosomes

Although the green filters represent proteins, as shown in Fig. 3, we utilized other channels as well as references. Therefore, we used all the filters as our inputs.

The data were provided as two versions of the same images, a scaled set of 512 × 512 PNG files and full-sized original images (a mix of 2048 × 2048 and 3072 × 3072 TIFF files). However, there are only full-sized images for our external data. To obtain more accurate prediction results, full-sized original images were adopted. Although the original images are of high quality, we must find a balance between model efficiency and accuracy. Therefore, we resized every image to 768 × 768 or 1024 × 1024 depending on its original size. We randomly crop 512 × 512 patches from 768 × 768 images (or cropping 768 × 768 patches from 1024 × 1024 images) when training-time augmentations before feeding the images to the models.

## Results and discussion

### Environments and settings

All experiments are implemented with Python 3.6 under the PyTorch framework [10]. To conduct a fair comparison, we reimplemented all methods, including all baselines and our methods, and optimized the relevant hyper-parameters and even structures for each method. As the classification task is multi-class and multi-label with 28 classes, the final output layers of all networks contain 28 units. All models are optimized by E-loss or other basic losses for comparison. Since there is no extra information, we cannot set $\alpha$ for each class in E-loss. Therefore, we tend to set $\alpha$ as a fixed value (such as 0.9, 0.99…) for all classes since the Vs are usually large enough. The batch sizes for the models depend on memory consumption and are set as large as possible to fit 8 NVIDIA GTX 1080 Ti GPUs. We use a stochastic gradient descent (SGD) with a default learning rate of 0.01, default weight decay of 1e−6 and, default momentum of 0.9 for the optimizer of all models.

### Trainings settings for different base models

#### DenseNet50

DenseNet [4] is an extraordinary architecture with good performance and a low number of parameters due to its feature reuse. We use the learning rates of [2e−3, 4e−3] to train for approximately 5 cycles until we find the best performance.

#### ResNet50

For modified ResNet50, cycle learning with a length of 4 epochs is used, and we apply the learning rates of [1e−2, 2e−2] to train for 3 or 4 cycles until we find the best performance. One buffering layer of 1024 units with a 0.5 dropout ratio is employed following the flattened ACP layer of 4096 units. We use 512 × 512 crops to feed the networks and adopt a batch size of 74 after several attempts.

#### InceptionV4

InceptionV4 has been widely applied. Although it improves the memory consumption problem on InceptionV3, it is still space-consuming. Therefore, we use a batch size of 48 with an input size of 512 × 512. The channel number of the encoder outputs is 1536, and it becomes 3072 after the ACP layer. We add one buffering layer with 1024 units for minimal information maintenance, followed by a 50% dropout.

#### GapNet-PL

This architecture has a relatively simple structure and a low number of parameters. To achieve the best performance, we add some convolutional blocks in the first step, thus passing more features to the second stage. A large input size of 768 × 768 is fed because we want to test the best capability on the dataset. The SGD with a momentum of 0.9 is kept as well as its original initial learning rate of 0.01. To avoid overfitting, the following regularization techniques are applied: L1 norm of 1e−7 and L2 norm of 1e−5, which is the same as the original settings. The dropout rate in the fully connected layers is still 30%, and we use a large batch size of 128.

#### ResNet101

Because we have enough data for feeding this large network, the model shall be solid. The alteration of ResNet101 is the same as that of ResNet50 except that the ACP layer is followed by two separate buffering layers with 4096 units, a 0.25 dropout ratio and 1024 units, a 0.5 dropout ratio.

#### SENet

The motivation of this network is explicitly to find the relationship between features and channels [11]. The squeeze-and-excitation mechanism helps the layers of the networks to “attend” the feature maps better and reduces the impact of unimportant features on the total results. Since there are many different kinds of proteins in our datasets, SENet could be helpful in weighting different filter channels based on the type of input data. We use [1e−3, 3e−3, 5e−3] as the learning rate to train the original SENet until divergence. A batch size of 80 is used.

### Evaluation and results

We use the F1-macro as our evaluating metric because it is a multi-class multi-labelled task. The equations are as follows:

1 $$\begin{array}{*{20}c} {R = \frac{TP}{{TP + FN}}} \\ \end{array}$$

2 $$\begin{array}{*{20}c} { P = \frac{TP}{{TP + FP}}} \\ \end{array}$$

3 $$\begin{array}{*{20}c} { F1 = \frac{2PR}{{P + R}}} \\ \end{array}$$

4 $$\begin{array}{*{20}c} { F1 \; macro = \frac{{\mathop \sum \nolimits_{i = 1}^{N} F1_{i} }}{N}} \\ \end{array}$$

where R denotes the recall and P represents precision. We use TP (true positive) and FN (false negative) to compute the recall scores and use TP and FP (false negative) for the precision scores. The F1 for each class is calculated by P and R. Because there are multiple classes, we averaged the F1 of each class and obtained an F1 macro score that represents the score of total classes.

Here, we use three tables to demonstrate the evaluation results of the proposed methods because the confusion matrix cannot be used. For each sample in the multi-labelled classification tasks, it is somewhat difficult to say which class is mistaken for another if there are two or more classes in the label of one sample. In Table 1, we can see that the proposed customized architectures help to greatly improve the identification accuracy (F1) of multi-class and multi-labelled protein images compared with the original backbones. Additionally, there is a large increase in F1 with hard example mining at the end of the networks. Therefore, this method should work well in most label-imbalanced situations. Additionally, we tried some more recent architectures in the backbone. Instead of using ResNet18, we adopted DenseNet50 with similar parameters but more layers, and it works better with or without modified architectures. We also tried SENet, which performed best in this task.

**Table 1:** Comparison of the model performance (F1) between original and modified ones

Model	Original	Customized	With hard sampler
ResNet50 (fold 0)	0.737	0.750	0.761
ResNet50 (fold 1)	0.729	0.734	0.752
ResNet50 (fold 2)	0.735	0.746	0.758
ResNet50 (fold 3)	0.731	0.741	0.749
ResNet50 (fold 4)	0.726	0.730	0.755
InceptionV4 (single fold)	0.736	0.749	0.756
SENet (single fold)	0.745	0.736	**0.771**
Dense50 (random fold 1)	0.732	0.738	0.741
Dense50 (random fold 2)	0.729	0.735	0.748
Dense50 (random fold 3)	0.732	0.741	0.754
ResNet101 (single fold)	0.742	0.758	0.760

The bold of “0.771” means that SENet with hard sampler performs best for the model performance (F1)

In Table 2, we know that sample duplication does exist in datasets, and E-loss works well together with other losses (focal) dealing with label imbalance problems based on the effective sample number of each class. The models can improve 1–2% in F1 with E-loss. Nevertheless, the modified SENet performs best in this stage and can outperform GapNet by approximately 2%.

**Table 2:** Scores of models with E-loss based on modified architectures (except GapNet)

Model with E-loss	Macro F1
ResNet50 (fold 0)	0.770
ResNet50 (fold 1)	0.766
ResNet50 (fold 2)	0.768
ResNet50 (fold 3)	0.758
ResNet50 (fold 4)	0.762
InceptionV4 (single fold)	0.767
SENet (single fold)	**0.782**
Dense50 (random fold 1)	0.758
Dense50 (random fold 2)	0.761
Dense50 (random fold 3)	0.766
ResNet101 (single fold)	0.778
GapNet-PL	0.765

The bold of “0.782” means that the modified SENet performs best in this stage

In Table 3, where TA denotes training-time optimization and TE denotes testing time, we can obtain the performance of all models based on Table 2, optimized with the proposed strategies (both training time and inference time). Although the effect may not be as significant as the previous methods, it can still help to increase approximately 1% in macro F1, making almost every model with single-fold data outperform the SOTA model GapNet-PL. Finally, we obtained some models with excellent performance using the proposed methods, which is proven in the tables above. The best score from the SENet backbone can reach up to 0.789 with only a single model, which is even 2.4% higher in the macro F1 score than that of the state-of-the-art model GapNet-PL.

**Table 3:** F1 of models with the proposed optimization strategies based on Table 2 (except GapNet)

Model	TA	TE	TA + TE
ResNet50 (fold 0)	0.774	0.772	0.774
ResNet50 (fold 1)	0.769	0.771	0.773
ResNet50 (fold 2)	0.773	0.769	0.775
ResNet50 (fold 3)	0.763	0.760	0.765
ResNet50 (fold 4)	0.765	0.763	0.767
InceptionV4 (single fold)	0.772	0.770	0.772
SENet (single fold)	**0.786**	0.783	**0.789**
Dense50 (random fold 1)	0.762	0.760	0.765
Dense50 (random fold 2)	0.765	0.763	0.766
Dense50 (random fold 3)	0.771	0.769	0.774
ResNet101 (single fold)	0.782	**0.784**	0.785
GapNet-PL	0.765	0.765	0.765

The bolds of “0.786, 0.784 and 0.789” mean that SENet, ResNet101 and SENet can reach up to the best macro F1 score for TA, TE and TA+TE, respectively

## Conclusion

In this work, we proposed an effective data processing method, as we made our training and testing datasets. When designing our network architectures, we proposed our customized layers, such as “buffering” layers and ACP layers, which made our networks more adaptive to the task. We proposed the hard sampler in our architecture for mining the samples of small classes better. We proposed a new loss to handle the label imbalance based on the sample effectiveness. We developed novel and effective optimization strategies in training and testing time. We designed group learning and cyclic learning with learning rate scheduling to solve the difficult optimization problem during training. For the test time post-process design, we proposed multi-threshold searching and multi-sized model ensembling.

Our evaluation results based on the test set split from the Human Protein Atlas dataset show the extraordinary performance of our methods. Our methods even outperformed the SOTA method of multi-labelled protein classification on the HPA dataset, GapNet-PL, by over 2% in the F1 score. This work demonstrates the great contributions and usefulness of our methods and networks for multi-class and multi-labelled high-throughput microscopy protein image recognition.

In future work, we may try different combinations of input channels instead of using all provided channel filters. We will adopt larger input crops if more computing resources are available.

## Methods

### Modified architectures and hard example mining

**Fig. 4:**
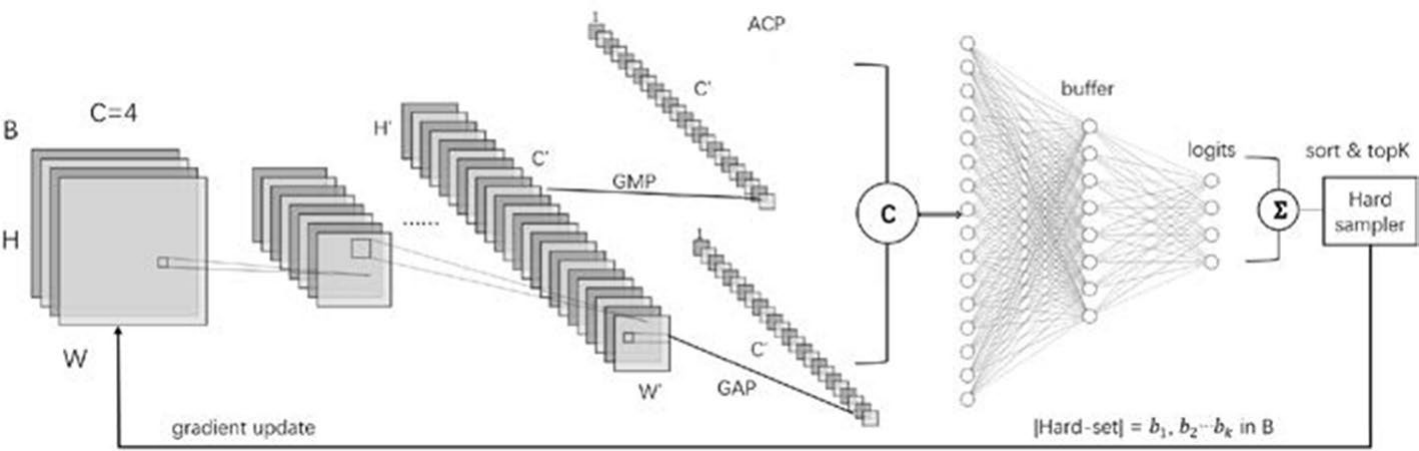
The novel network architecture with 4-channel inputs and ACP layer, as well as Hard Sampler for HEM. Full explanation of GAP/GMP/ACP is available at the end of the article

We basically adopt some typical classification backbones, such as ResNet or InceptionNet, with our novel modifications. We improve the networks with input layers, connection parts and head parts to make our models more accurate and adaptive. Figure 4 shows the novel network architecture we proposed. The encoders are collected mostly from the widely used classifiers because the latter have been proven effective in many tasks. We replace the 3-channel input layers with 4 channels, as we want to use all filter channels.

We drop the GAP (global average pooling) layers at the beginning of other typical heads in the connection parts because high-throughput microscopy images are often related to high resolution and various sizes. Instead, we propose and build an ACP layer by concatenating two kinds of pooling layers, an adaptive average pooling layer and an adaptive max-pooling layer, in the channel dimension. An adaptive average pooling layer applies GAP (global average pooling) over an input signal composed of several input planes, while an adaptive max-pooling layer applies GMP (global max-pooling). By combining them, we can acquire both advantages. The output size is H × W for any input size. The number of output features is equal to the number of input planes. This strategy allows us to decide what output dimensions we want instead of choosing the input dimensions to fit a desired output size. Therefore, we set the H and W to be 1. Regardless of the size of the input images, this layer will act as an adaptive global pooling. We utilize and concatenate both types of pooling layers to provide the model with the information of both methods and to improve performance.

In the head parts, we add some “buffering layers” to prevent rapid feature information loss. Usually, the channel amounts would be large after the encoder; if we shrink the channel number to the target channel number as classifiers usually do, many features will be lost in this fully connected process. Therefore, we add one or two middle FC layers with decreasing numbers of neurons to maintain the feature information.

Since there is extreme label imbalance in datasets, the samples of large classes are dominant in the final losses. The small samples, on the contrary, are hard to learn. After several backward iterations of the gradient, the weights are trained to predict normal samples better, and losses of those samples tend to be small. Here, we introduce a method that can learn the small classes better based on the final losses of each batch. We designed a hard sampler to first sort the samples in this batch by their losses (as shown in Fig. 4). Then, we select the top K losses, which are basically generated by small classes, from B samples. Therefore, the majority classes contribute almost nothing during backpropagation, while the minority classes dominate the learning process, making it easier to learn the head examples. These novel modifications all worked in the experiments because they helped the classifiers be more accurate and adaptive to this kind of task, especially for the learning of hard samples in small classes.

### E-loss with label imbalance and effectiveness

As there is severe label imbalance in the datasets, we adopt a weighted loss function for different classes based on their numbers. However, there are more similarities among samples in majority classes, which would cause duplication or overlapping in feature space. Therefore, technically, assume we have n samples, and each takes 1 volume in the feature space area. It is not hard to find that the total volume V must be less than n because there may be overlap among every group of samples. The larger the class is, the more duplications may exist in this group. Therefore, it is illogical to simply use the sample number as the loss weight for that class. We should use the effective numbers to do that.

**Fig. 5:**
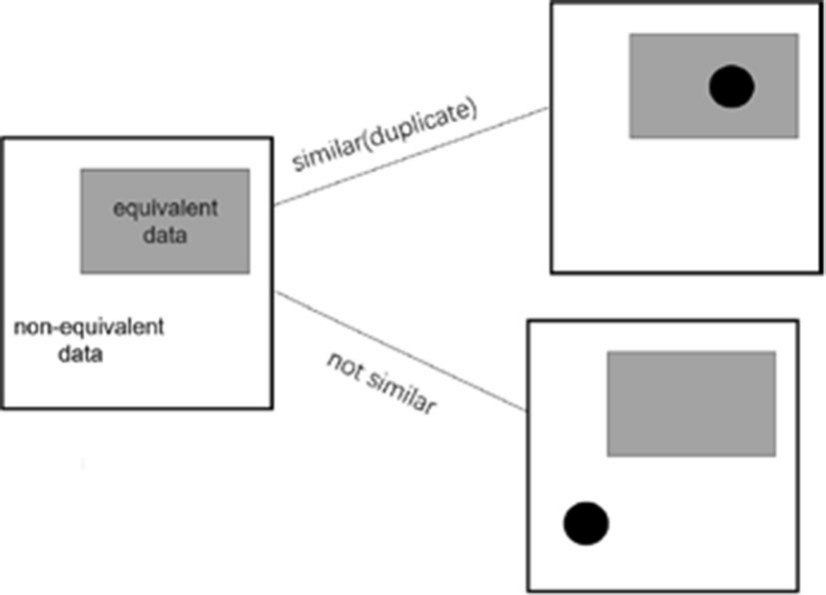
The current sample could be overlapped with previous samples by possibility p, or not by possibility 1 − p. Dark cycle means some random sample in this iteration, and the gray rectangle denotes the feature space of the existing samples obtained before

Now, we have to calculate the expectation number (volume) ${Q}_{n}$ with n current samples. Assuming the probability that the newly added sample can be represented by other existing samples is p, the probability is 1 − p when adding a brand-new sample in the feature space. Figure 5 demonstrates that the current sample could overlap with the previous samples by possibility p or not overlap by possibility 1 − p. Obviously, $p = Q_{n - 1}$/V. Therefore, we have the following equation:

5 $$\begin{array}{*{20}c} { Q_{n} = p*Q_{n - 1} + \left( {1 - {\text{p}}} \right)*\left( {Q_{n - 1} + 1} \right) = \frac{V - 1}{V}Q_{n - 1} + 1} \\ \end{array}$$

Apparently, $Q_{1}$ = 1. Let $\frac{V - 1}{V}$ = $\alpha$, and we can obtain $Q_{n}$ = $\frac{{1 - \alpha^{n} }}{1 - \alpha }$ from induction. When V = 1, $\alpha$ = 0, $Q_{1}$ = 1. When ${\text{V}} \to \infty$, $\alpha \to 1$, $Q_{n} \to n$. Assume the batch number is B, and they belong to classes $c_{1}$ and $c_{2} \ldots c_{k}$, respectively; then, we have:

6 $$\begin{array}{*{20}c} { EL = \mathop \sum \limits_{c} \frac{1}{{Q_{{n_{c} }} }}{\text{L}}\left( {p_{c} ,{ }y_{c} } \right) = { }\mathop \sum \limits_{c} \frac{1 - \alpha }{{1 - a^{{n_{c} }} }}{\text{ L}}\left( {p_{c} ,{ }y_{c} } \right)} \\ \end{array}$$

where ${\text{L}}\left( {p_{c} ,{ }y_{c} } \right)$ denotes the loss (any loss function such as BCE) between the ground truth and predictions, and $n_{c}$ denotes the sample number of class C. Therefore, the weight $\frac{1}{{n_{c} }}$ of each class is replaced by $\frac{1}{{Q_{{n_{c} }} }}$ from the proposed E-loss, which contributes greatly to the final results.

### Optimization strategies

Although we may have designed good architectures, we discovered that it is rather challenging to train on this dataset, and we cannot obtain an ideal result. Therefore, we design more effective and efficient optimization strategies at training and testing times.

### Training-time optimization strategies

When choosing the training strategies, we divide our networks into 2 different layer groups with distinct learning rates during training. Moreover, we adopted cyclic learning with cosine annealing and learning rate scheduling during gradient descent.

The first learning rate group contains mainly the encoder layers, and the second group will usually contain the heads. We apply distinct learning rates to different layer groups. Building CNNs based on pre-trained weights usually results in better performance than building CNNs from scratch because pre-trained weights have already learned many object features from other data, which means that they have some prior knowledge. The earlier the layers lie, the more basic features they contain. The primary features do not need to change because they are very similar. It is necessary to change most weights of the head for a new task because they are newly initialized and present more high-level object features. Because the images in the HPA datasets are quite different from those in ImageNet, which only include normal and daily pictures, we decrease the learning rate of the first layer group (encoder part) by only 2–3 times compared with that of the second group. Therefore, the models are trained with [lr/3, lr] or [lr/4, lr/2, lr], where lr denotes the learning rate.

When choosing the starting learning rates, we no longer attempt the learning rate from a larger learning rate to a small one. We first find the optimal initial learning rate with a relatively low learning rate (approximately 1e−5). Then, we gradually increase the learning rate exponentially with each batch, and the loss is recorded in an array for each learning rate at the same time. The current optimal learning rate is the value found where the learning rate is highest yet the loss is still decreasing.

**Fig. 6:**
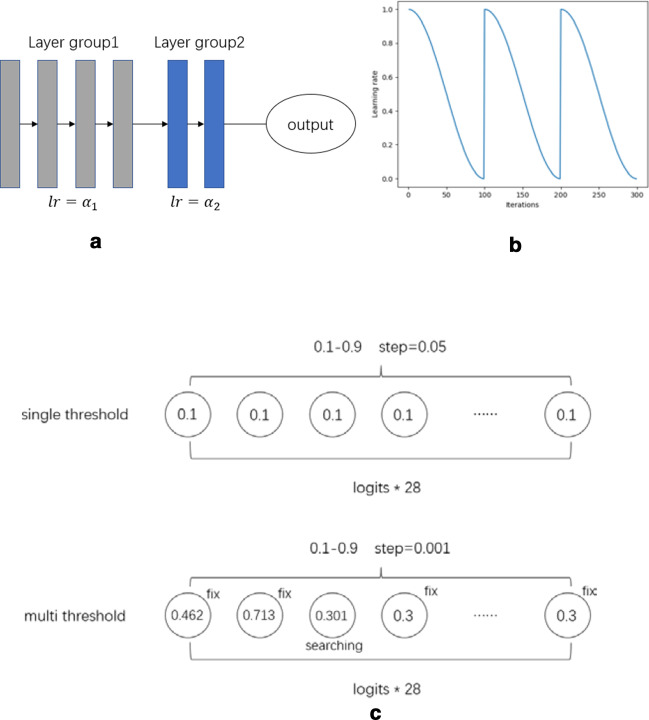
**a** The network is divided into 2 layer groups with separate learning rates. **b** An example of cycle learning with cosine annealing and cycle repetition (lr restart). **c** The single (above) and multi-threshold (below) in the post optimization process based on greedy searching

Because we have our optimal starting learning rate, we adopted cycle learning with learning rate scheduling to train our models (see Fig. 6b). Cycle learning, inspired by Leslie Smith’s work [12], contains two key factors: cosine annealing and cycle repetition. During the training process, the total loss of the architecture should be increasingly closer to the minimum (local or global). However, it is often difficult to converge as the loss approaches its minimum value. Cosine annealing solves the problem by decreasing the learning rate following the mode of cosine function with each batch of data. We start at a high-level learning rate and decrease the learning rate based on a cosine function. We found that this mode of learning rate decrease works very well with the convergence problem in this task. Moreover, it is very likely for loss to be trapped at its local minimum instead of at the global minimum during training. Therefore, if we increase the learning rate suddenly, the current loss may find its way towards the global minimum by “jumping” out of the local minimum with a larger step. We reset the learning rate each time the learning rate drops to its minimum value by cosine annealing, and we call that a “cycle”. We repeat the process every time one cycle is performed until the loss hardly decreases.

### Testing-time optimization strategies

Additionally, we proposed a novel and effective method for choosing the thresholds for each class. During training, we simply used 0.5 as the threshold value of output logits for 28 classes. However, at test time, 0.5 may not be the best value for each class. The model responds very strongly to common classes because they have more training samples than the others. Therefore, the probability scores of these classes are more likely to be higher than 0.5, even approaching 1.0. The rare classes, in contrast, tend to obtain much smaller scores, even close to 0. We first find the optimal single threshold for all classes by searching the threshold from 0.1 to 0.9 by a step of 0.05 and further obtain an optimal validation F1 score at one threshold. Then, we use this value, equal to 0.3, as the initial value for each class. We start from the first class and fix the thresholds of other classes, finding and choosing the local optimal value with which the highest score can be achieved on the validation set. Next, we move on to the second class and do the same. Obviously, this is a “greedy” process (see Fig. 6c). After we finish all 28 classes, the process is performed, and it works well in the experiments.

For ensemble learning, we used cross-validation for each backbone model. Finally, we use models with different input sizes and different architectures. The performance improved slightly on single models by averaging the advantages of various models with different input sizes.

## Data Availability

The datasets used in our experiments are available at https://www.kaggle.com/c/human-protein-atlas-image-classification. The images included in this study are available in the HPA Cell Atlas (https://www.proteinatlas.org).

## Abbreviations

HPA: Human Protein Atlas
CNNs: Convolutional neuron networks
TA: Training-time optimization
TE: Testing-time optimization
GAP: Global average pooling
GMP: Global max pooling
ACP: Adaptive concatenate pooling
HEM: Hard example mining
